# Analytical fleet-sizing method for the Open Platform for Innovation in Logistics (OPIL)

**DOI:** 10.1038/s41598-026-46148-y

**Published:** 2026-04-09

**Authors:** Ladislav Körösi, František Duchoň

**Affiliations:** Institute of Robotics and Cybernetics, Faculty of Electrical Engineering and Information Technology of the Slovak University of Technology in Bratislava, Ilkovičova 3, 84104 Bratislava, Slovakia

**Keywords:** Logistics, Material flow, Agent, Optimization, IoT, Docker, Engineering, Mathematics and computing

## Abstract

This manuscript presents an analytical fleet-sizing module developed for the Open Platform for Innovation in Logistics (OPIL) within the European Horizon 2020 project “Better Factory“. The module provides a deterministic capacity estimation method for calculating the required number of logistics agents, such as automated guided vehicles, forklifts, or human operators, needed to satisfy predefined material flow demands under steady-state conditions. The approach extends a classical analytical framework by incorporating matrix-based representations of material flows and distances together with agent-specific operational parameters. It is designed to support early-stage logistics planning and system scaling, particularly in small and medium-sized enterprises, where accessible and transparent decision-support tools are essential. The module is implemented as an independent application and its applicability is demonstrated through two industrial case studies involving material transport and inspection tasks. The results illustrate how the proposed analytical framework can support practical decision-making in logistics system design within clearly defined operational assumptions.

## Introduction

Modern production systems increasingly adopt Industry 4.0 principles, emphasizing digitalization, data integration and continuous system improvement. While substantial research attention has been directed toward optimizing production technologies, material handling and internal logistics remain critical components of manufacturing performance. Even though precise values vary across industries, transportation and material handling represent a significant portion of logistics costs and operational time^1,2^. Inefficient logistics can therefore substantially affect productivity, resource utilization and investment decisions.

Recent global supply chain disruptions, labor shortages, and increasing operational complexity have further highlighted the need for improved transparency and analytical support in logistics systems^3–5^. Digitalization of material flows, combined with data-driven methods such as discrete event simulation and statistical analysis enables improved bottleneck identification, resource planning and system evaluation^6–8^. However, many available solutions require specialized expertise or extensive modeling effort, which may limit their accessibility, particularly for small and medium-sized enterprises (SMEs). Previous research has shown that SMEs often face significant challenges in adopting advanced manufacturing and simulation technologies due to limited technical expertise, lack of skills and resource constraints, indicating that accessible analytical methods can better support early planning needs of such firms^9–11^.

Commercial platforms such as Tecnomatix^®^, SIMIT^®^, Visual Components^®^, and Delmia^®^ provide advanced simulation and optimization capabilities^12–14^. In practice, however, logistics planning frequently relies on iterative trial-and-error procedures or complex computational methods that may be difficult to interpret or implement without expert knowledge. Open frameworks such as the OPIL^15^ aim to increase accessibility by providing modular and interoperable tools for logistics applications.

Within the research literature, logistics optimization has predominantly focused on routing, scheduling, fleet management and task assignment problems^16^. Advanced approaches include heuristic and metaheuristic algorithms, artificial neural networks, response surface methodology and other data-driven techniques^17–20^. While these methods address path optimization and agent utilization, comparatively less attention has been devoted to transparent analytical methods for estimating the required number of logistics agents needed to satisfy predefined material flows under steady-state conditions. Existing analytical solutions are often application-specific or limited to particular agent types.

This study addresses this gap by presenting a deterministic analytical fleet-sizing method derived from and extending Groover’s classical approach^21^. The proposed framework incorporates matrix-based representations of material flows and distances and integrates agent-specific operational parameters such as speed, loading and unloading times, availability, traffic influence and worker efficiency. Rather than formulating a formal optimization problem with explicit objective functions and constraints, the method provides a structured capacity estimation procedure for determining the number of agents required to satisfy given flow demands under clearly defined assumptions.

The methodology has been implemented as an independent module within the OPIL ecosystem and can operate either as a standalone application or as part of a larger logistics architecture. Its applicability is demonstrated through two industrial case studies involving material transport and inspection tasks within the Better Factory project. The objective is not to replace advanced simulation-based approaches, but to provide an accessible and transparent analytical decision-support tool suitable for early-stage planning, system scaling and investment assessment, particularly in SME environments.

The remainder of the paper describes the OPIL framework, presents the analytical methodology and implementation and evaluates the approach through industrial use cases. Assumptions, operational scope and limitations of the proposed method are explicitly discussed to ensure appropriate interpretation of the results.

The methodological contribution of this work lies not in introducing a fundamentally new optimization paradigm, but in providing a structured and practically applicable analytical formulation that integrates multiple real-world operational factors within a unified deterministic framework. In contrast to classical formulations, the proposed method enables direct capacity estimation using matrix-based flow representation, incorporates availability, traffic and human-efficiency effects in a consistent manner, and allows straightforward scenario-based analysis without requiring simulation or iterative optimization procedures.

## Open platform for innovation in logistics

The OPIL is an open and modular framework designed to support logistics applications in Industry 4.0 environments, particularly within SMEs^1^. It provides interoperable components for task planning, path planning, simulation and system integration, enabling the development and evaluation of logistics solutions within heterogeneous production systems.

**Fig. 1:**
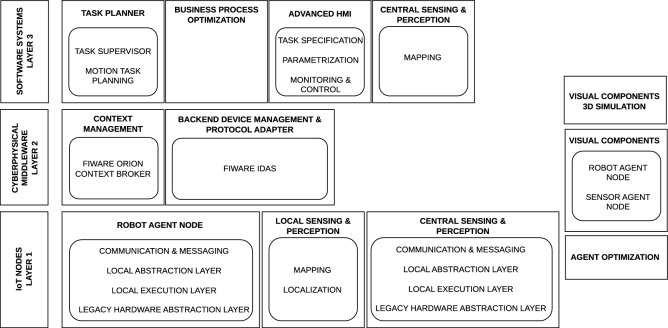
Architecture of OPIL.

OPIL follows a layered architecture that connects physical devices (e.g., mobile robots, AGVs, forklifts, sensors and human–machine interfaces) with middleware services and higher-level software modules (Fig. 1). Communication between components is based on the FIWARE ecosystem and the Next Generation Service Interface (NGSI), enabling standardized data exchange and integration with external IT systems such as ERP and warehouse management systems. The architecture supports extensibility through open-source technologies, including ROS-based robotic modules and database-backed services.

Within this ecosystem, the Agent Optimization module was developed as a decision-support component for estimating the required number of logistics agents. Although designed to integrate with OPIL through standardized data interfaces (MongoDB-based storage and structured input matrices), the module operates as an independent Docker-based application. This design ensures reproducibility, portability and the possibility of deployment either within the OPIL framework or as a standalone analytical tool in other logistics environments.

The module interacts with the surrounding system only through defined input parameters (distance matrices, flow-rate matrices, and agent characteristics) and produces structured outputs representing the estimated number of required agents. By decoupling the analytical method from the broader platform architecture, the solution maintains flexibility while preserving compatibility with larger logistics infrastructures.

In this paper, the focus is placed on the analytical methodology and its validation, while the OPIL framework provides the technological context in which the module can be deployed.

## Agent sizing method

This section presents the analytical fleet-sizing methodology and its implementation. The proposed module estimates the number of logistics agents required to satisfy predefined material flow demands under steady-state conditions. Although originally developed within the OPIL ecosystem, the module operates as an independent Docker-based application and can be deployed either standalone or integrated through a standardized data interface (MongoDB-based structured input).

### Methodology

The proposed method extends the analytical framework introduced by Groover^21^ and provides a deterministic capacity estimation procedure. The objective is to determine the number of agents required to satisfy given material flow rates within a defined planning horizon. In this study, all quantities are expressed per hour, however, the formulation remains valid for any consistent time unit, provided that flow rates, speeds and times are defined accordingly.

#### Input parameters

The model requires the following agent parameters:

- $v_c$ — average agent travel speed [m/min], $v_c> 0$
- $T_L$ — loading time per trip [min], $T_L> 0$
- $T_U$ — unloading time per trip [min], $T_U> 0$
- *c* — agent transport capacity [pieces/trip], $c> 0$
- *A* — availability factor (proportion of operational time), $A \in (0,1]$
- $F_t$ — traffic factor accounting for congestion effects, $F_t \in (0,1]$
- $E_w$ — operator efficiency factor, $E_w \in (0,1]$

The agent speed represents an average operational speed reflecting practical constraints. Loading and unloading times may be obtained from measurements or historical production data. The availability factor *A* represents the fraction of scheduled time during which the agent is operational. The traffic factor $F_t$ captures systematic performance losses due to congestion or shared workspace interactions. The efficiency factor $E_w$ accounts for operator-related variability and is set to 1.0 for fully autonomous agents.

#### Flow and distance representation

Material flows are represented by an $N \times N$ flow matrix $F_{ij}$, where each element denotes the average flow rate between stations *i* and *j* expressed in pieces per hour (pcs/hr). The distance matrix $D_{ij}$ defines the physical travel distance between stations in meters. Negative entries in the flow matrix indicate predefined empty return paths. The corresponding distances are taken from the same distance matrix $D_{ij}$, which allows modeling of closed transport loops using a consistent distance definition.

#### Transport demand and cycle time

The material flow matrix $F_{ij}$ expresses the required transport demand in pieces per hour between stations *i* and *j*. In the implemented procedure, the total number of required deliveries per hour is computed as the sum of all positive flow entries:

1 $$\begin{aligned} w = \sum _{i,j \, | \, F_{ij}> 0} F_{ij}. \end{aligned}$$

Thus, *w* represents total number of required delivery cycles per hour. The agent capacity *c* is incorporated in the calculation of the average loaded distance. The average loaded distance per delivery is computed as

2 $$\begin{aligned} L_d = \frac{\sum _{i,j \, | \, F_{ij}> 0} F_{ij} D_{ij}}{c \sum _{i,j \, | \, F_{ij}> 0} F_{ij}}, \end{aligned}$$

which represents the mean loaded travel distance per delivery cycle adjusted by the transport capacity *c*. The empty travel distance per delivery cycle is obtained from predefined negative flow entries. In the implemented model, each negative entry represents a single empty return movement and the corresponding empty distance is computed as

3 $$\begin{aligned} L_e = \sum _{i,j \, | \, F_{ij} < 0} D_{ij}. \end{aligned}$$

A negative flow entry represents a predefined empty return movement. For example, $F_{ij} = -1$ indicates one empty return trip along the link from station *i* to station *j*, with the corresponding distance taken from $D_{ij}$. The overall delivery cycle time is then expressed as

4 $$\begin{aligned} T_C = T_L + \frac{L_d}{v_c} + T_U + \frac{L_e}{v_c}, \end{aligned}$$

where $T_L$ and $T_U$ denote loading and unloading times and $v_c$ is the average agent speed. The quantity $T_C$ represents the time required to complete a single delivery cycle. The total workload per hour is therefore

5 $$\begin{aligned} WL = w \, T_C, \end{aligned}$$

which represents the total required agent operating time per hour.

#### Available time and required number of agents

The available time per hour per agent is given by

6 $$\begin{aligned} AT = 60 A F_t E_w, \end{aligned}$$

where *A* is the availability factor, $F_t$ is the traffic factor and $E_w$ is the operator efficiency. The required number of agents is determined as

7 $$\begin{aligned} AN = \frac{WL}{AT}. \end{aligned}$$

Since fractional agents are not physically meaningful, the result is rounded up to the nearest higher integer:

8 $$\begin{aligned} AN_{\text {final}} = \lceil AN \rceil . \end{aligned}$$

Rounding upward ensures that the computed fleet size satisfies the required transport demand without under-capacity and provides a conservative estimate suitable for planning purposes.

#### Calibration of traffic and efficiency factors

The parameters $F_t$ (traffic factor) and $E_w$ (operator efficiency) represent aggregated performance modifiers and therefore require careful operational definition. In practice, $F_t$ can be estimated empirically as the ratio between the theoretical travel time under free-flow conditions and the observed average travel time obtained from logged operational data, i.e.

9 $$\begin{aligned} F_t = \frac{T_{\text {theoretical}}}{T_{\text {observed}}}. \end{aligned}$$

Here, $T_{\text {theoretical}}$ is computed from nominal route length and average free-flow speed, while $T_{\text {observed}}$ is derived from historical measurements reflecting congestion, route intersections or shared workspace interactions. Similarly, the operator efficiency factor $E_w$ represents the influence of human performance variability on loading and unloading operations. In contrast to $F_t$, which primarily affects travel performance, $E_w$ reflects the deviation of actual handling time from nominal or planned handling time due to ergonomic constraints, fatigue, lifting conditions or workforce allocation policies. In practice, $E_w$ can be estimated as the ratio between the nominal loading/unloading time used in planning and the observed average handling time measured during operation. When loading and unloading are fully automated and independent of human intervention, $E_w$ is set to 1.0. This definition ensures that human-related variability is incorporated without redefining the structural cycle-time formulation. These types of empirical correction factors are commonly used in industrial engineering practice to account for deviations between nominal and observed performance, particularly in environments where direct modelling of congestion and human variability is not feasible. In practical applications, these parameters are typically identified from historical operational data or short-term measurements conducted under representative working conditions.

#### Sensitivity considerations

Since the required number of agents is inversely proportional to the product $A F_t E_w$, the model exhibits linear sensitivity with respect to these parameters:

10 $$\begin{aligned} AN \propto \frac{1}{A F_t E_w}. \end{aligned}$$

Consequently, a relative decrease of $x\%$ in either $F_t$ or $E_w$ results in an approximately $x\%$ increase in the required agent capacity, assuming all other parameters remain constant. This proportional behavior provides predictable and interpretable system response. In typical industrial scenarios where $F_t$ and $E_w$ vary within a limited range (e.g., 0.9–1.0), the resulting variation in the required fleet size remains moderate. The monotonic and analytically transparent dependency enhances robustness and allows practitioners to perform rapid scenario testing by adjusting these parameters according to anticipated congestion levels or workforce variability.

#### Model assumptions and scope

The proposed analytical method operates under the following assumptions:

- Steady-state operation with deterministic average flow rates.
- Average constant agent speed.
- Predefined transport routes represented by the distance matrix.
- No explicit modeling of queueing or dynamic task scheduling.
- Interaction effects, congestion, and operational variability are aggregated into the parameters $F_t$, *A*, and $E_w$.

The method is therefore intended for capacity planning and early-stage system design rather than detailed dynamic simulation. In environments with highly stochastic flows or complex routing conflicts, complementary simulation-based validation may be appropriate.

### Implementation

The Agent Optimization module was implemented as an independent Docker-based service to ensure portability, reproducibility, and platform independence. The computational core is encapsulated within a Docker container that executes the analytical sizing algorithm described in Section “Methodology”. This design enables deployment either as a standalone microservice or as part of a broader logistics ecosystem such as OPIL.

The service operates using a MongoDB-based data interface. Input parameters, including agent characteristics and the corresponding distance and flow-rate matrices are stored as structured documents within the database. Each optimization request is uniquely identified by a request identifier. Upon execution, the service retrieves the associated document, performs the analytical computation and writes the resulting fleet-size values back to the database. The database instance may run either locally on the host system or within a separate container enabling flexible deployment scenarios.

The required input data include the number of stations, the distance matrix, the flow-rate matrix (expressed in pcs/hr), agent parameters ($v_c$, $T_L$, $T_U$, *c*, *A*, $F_t$, $E_w$).

Matrices are encoded as structured numerical arrays within the database representation. Negative entries in the flow matrix are used solely to indicate predefined return paths, as described in Section “Methodology”.

**Fig. 2:**
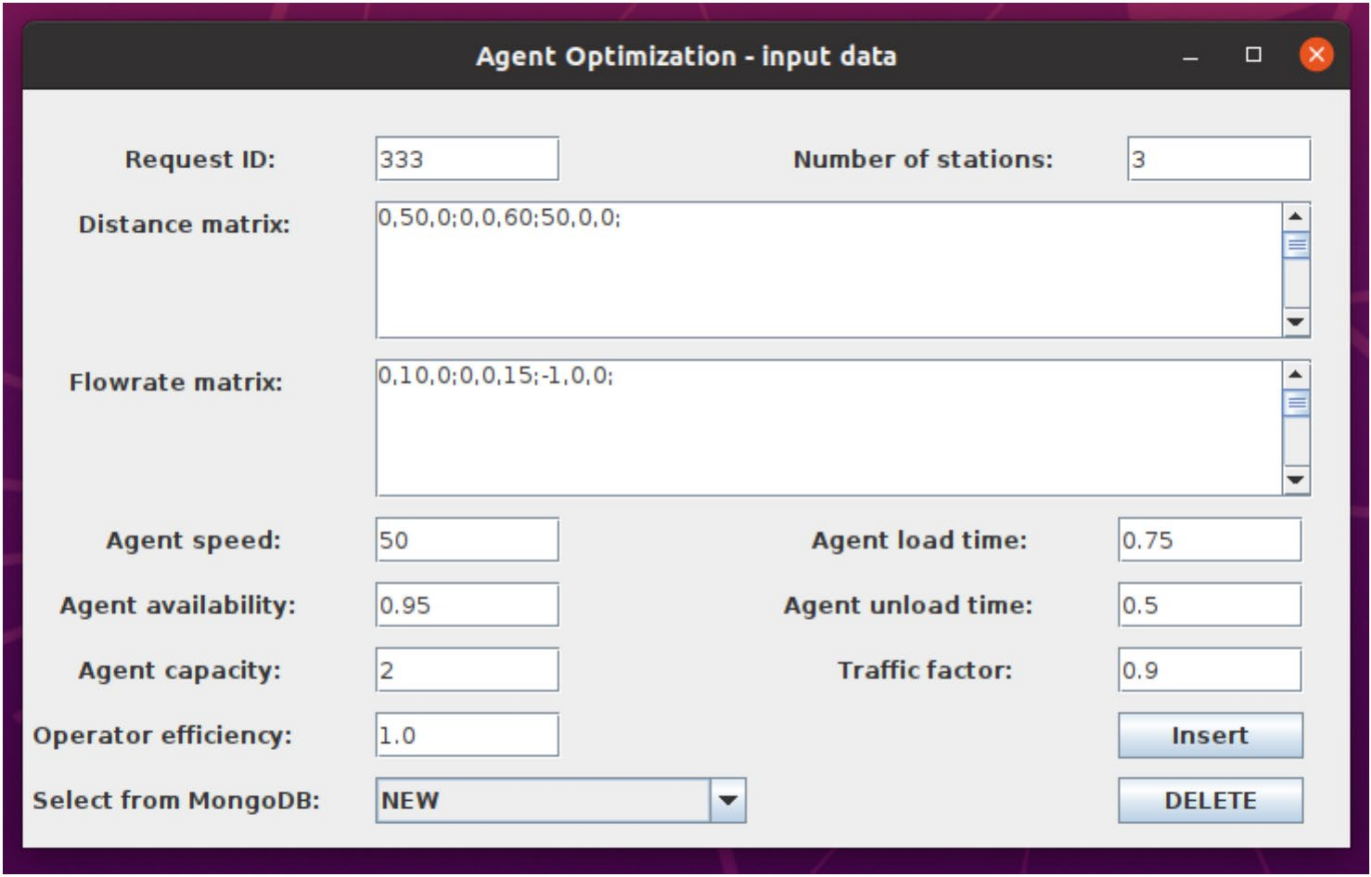
Human machine interface for Agent Optimization.

Two interaction modes are supported. The first enables direct database interaction through a graphical Human–Machine Interface (HMI) developed in Java (2). The HMI communicates with MongoDB via the official MongoDB Java Driver and includes input validation mechanisms that verify formatting consistency, dimensional compatibility of matrices, admissible parameter ranges, and uniqueness of request identifiers. The second mode allows external applications to programmatically submit optimization requests through standard database communication protocols, enabling integration with ERP, MES, or digital twin systems (Fig. 2).

The complete source code of the Agent Optimization service is publicly available in the Better Factory application repository (https://github.com/ramp-eu/Agent_Optimisation_Service)^22^. This ensures full reproducibility of the computational results presented in this study. The complete distance and flow-rate matrices used in the case studies are provided in the Supplementary Information.

## Examples

### Example 1 - SMARTENVELOPE

#### Industrial context and transport structure

The SMARTENVELOPE Knowledge Transfer Experiment (KTE) was conducted at Plast-Farb, a Polish manufacturer of security envelopes. The objective was to determine the required number of forklifts for internal material transport under steady-state production conditions.

Three dominant transport routes were identified:

1. Transport of raw material (foil rolls) from warehouse to production machines.
2. Transport of finished pallets from production machines to the stretch-wrapping station.
3. Transport of wrapped pallets back to the warehouse.

A 2D factory model was used to determine transport distances between stations, while a simplified 3D layout in OPIL library supported route verification. The analysis was performed under steady-state assumptions using hourly averaged material flows.

#### Input data and operational parameters

Material flows were derived from ERP data representing pallet production per shift and converted to average hourly flow rates. The resulting $N \times N$ flow rate matrix (pallets/hour) is given in Tab. 1 and the corresponding distance matrix (meters) in Tab. 2 in the supplementary attachment.

The forklift parameters were:

- Agent speed: $v_c = 35$ m/min
- Loading time: $T_L = 0.4$ min
- Unloading time: $T_U = 0.5$ min
- Capacity: $$c = 1$$ pallet
- Availability: $$A = 0.7$$
- Traffic factor: $$F_t = 0.5$$
- Operator efficiency: $$E_w = 0.7$$

All calculations are performed per hour. Capacity $c=1$ reflects one pallet per trip.

#### Analytical fleet sizing using proposed model

From the flow and distance matrices, the average loaded and empty travel distances were computed according to the methodology described in Section "Agent Sizing Method". The intermediate derived quantities representing delivery cycles were:

$$\begin{aligned} w = 10.222 \ \text {pallets/hour}, \quad L_d = 40.30 \ \text {m}, \quad L_e = 28.76 \ \text {m}. \end{aligned}$$

The resulting average cycle time per delivery was:

$$\begin{aligned} T_C = 2.912 \ \text {min}. \end{aligned}$$

The total workload per hour is:

$$\begin{aligned} WL = 29.766 \ \text {min/hour}. \end{aligned}$$

The effective available time per forklift per hour is:

$$\begin{aligned} AT = 60 \cdot A \cdot F_t \cdot E_w = 60 \cdot 0.7 \cdot 0.5 \cdot 0.7 = 14.7 \ \text {min/hour}. \end{aligned}$$

The required number of forklifts is therefore:

$$\begin{aligned} AN = \frac{WL}{AT} = \frac{29.766}{14.7} = 2.025. \end{aligned}$$

Since fractional agents are infeasible in practice, the result is rounded up:

$$\begin{aligned} AN_r = 3. \end{aligned}$$

#### Baseline comparison with classical analytical fleet sizing

For comparison, a classical deterministic fleet sizing without correction factors (i.e., assuming $A = F_t = E_w = 1$) yields:

$$\begin{aligned} AT_{baseline} = 60 \ \text {min/hour}, \end{aligned}$$

$$\begin{aligned} AN_{baseline} = \frac{29.766}{60} = 0.496. \end{aligned}$$

This baseline approach would incorrectly suggest that a single forklift is sufficient. The difference illustrates the importance of incorporating availability, traffic and operational efficiency factors. Without these correction factors, fleet size would be significantly underestimated.

#### Sensitivity analysis

To evaluate robustness, key parameters were varied individually while keeping other parameters constant.

**Traffic factor variation**

- $$F_t = 0.4$$ $$\Rightarrow$$ $$AT = 11.76$$ min/hour $$\Rightarrow$$ $$AN = 2.53$$
- $$F_t = 0.6$$ $$\Rightarrow$$ $$AT = 17.64$$ min/hour $$\Rightarrow$$ $$AN = 1.69$$

**Availability variation**

- $$A = 0.6$$ $$\Rightarrow$$ $$AT = 12.6$$ min/hour $$\Rightarrow$$ $$AN = 2.36$$
- $$A = 0.8$$ $$\Rightarrow$$ $$AT = 16.8$$ min/hour $$\Rightarrow$$ $$AN = 1.77$$

**Speed variation** ($$\pm 10\%$$)

The cycle time consists of fixed handling components and a travel component:

$$\begin{aligned} T_C = T_L + T_U + \frac{L_d + L_e}{v_c}. \end{aligned}$$

Only the transport term depends on $v_c$, while loading and unloading times remain constant. Therefore, the relationship between $T_C$ and $v_c$ is nonlinear but monotonic.

- $$v_c = 36$$ m/min ($$-10\%$$) $$\Rightarrow$$ $$T_C = 3.08$$ min $$\Rightarrow$$ $$AN = 2.14$$
- $$v_c = 44$$ m/min ($$+10\%$$) $$\Rightarrow$$ $$T_C = 2.78$$ min $$\Rightarrow$$ $$AN = 1.93$$

The analysis shows monotonic and proportional behavior consistent with the analytical formulation:

$$\begin{aligned} AN \propto \frac{1}{A F_t E_w}. \end{aligned}$$

The rounding boundary between two and three forklifts lies near $AN = 2$. Moderate parameter variations do not cause unstable behavior, confirming robustness of the sizing model under realistic fluctuations.

**Combined Parameter Variation (Scenario-Based Bounds)**

To assess the combined influence of key operational parameters, two representative scenarios were evaluated reflecting optimistic and conservative operating conditions.

In the optimistic scenario, nominal parameter values were assumed with no congestion or efficiency losses ($F_t = 1.0$, $E_w = 1.0$), while maintaining the original availability $A = 0.7$ and agent speed $v_c = 40\ \mathrm {m/min}$. The resulting available time per agent is:

11 $$\begin{aligned} AT = 60 \cdot A \cdot F_t \cdot E_w = 60 \cdot 0.7 \cdot 1.0 \cdot 1.0 = 42\ \mathrm {min/hour}. \end{aligned}$$

The required number of agents is therefore:

12 $$\begin{aligned} A_N = \frac{WL}{AT} = \frac{29.766}{42} = 0.709. \end{aligned}$$

After rounding:

13 $$\begin{aligned} A_{N,\textrm{final}} = 1. \end{aligned}$$

In the conservative scenario, reduced performance due to congestion and operator variability was considered ($F_t = 0.5$, $E_w = 0.5$), together with reduced effective speed $v_c = 32\ \mathrm {m/min}$ ($-20\%$). The reduced speed increases the transport component of the cycle time, resulting in an increased workload:

14 $$\begin{aligned} T_{req} \approx 32.7\ \mathrm {min/hour}. \end{aligned}$$

The corresponding available time per agent is:

15 $$\begin{aligned} AT = 60 \cdot A \cdot F_t \cdot E_w = 60 \cdot 0.7 \cdot 0.5 \cdot 0.5 = 10.5\ \mathrm {min/hour}. \end{aligned}$$

The required number of agents is therefore:

16 $$\begin{aligned} A_N = \frac{32.7}{10.5} = 3.11. \end{aligned}$$

After rounding:

17 $$\begin{aligned} A_{N,\textrm{final}} = 4. \end{aligned}$$

These results define practical lower and upper bounds for fleet sizing under simultaneous parameter variations, ranging from 1 to 4 forklifts. The analytical structure of the model allows such combined scenarios to be evaluated directly, as the required number of agents depends multiplicatively on the parameters *A*, $F_t$, and $E_w$. This provides a simple and interpretable mechanism for assessing robustness without requiring simulation-based analysis.

#### Operational confirmation

At the time of analysis, three forklifts were available in the facility. However, one unit served primarily as backup for maintenance and breakdown scenarios. Operationally, two forklifts are actively used during steady-state production. The analytical result ($AN = 2.025$) corresponds closely to the observed operational requirement of two active forklifts, while the rounding rule recommends three units to ensure service continuity. The result is therefore operationally consistent with industrial practice.

#### Quantitative consistency assessment

To further evaluate the consistency of the analytical estimation with observed industrial deployment, a quantitative utilization analysis was performed.

The analytically predicted continuous fleet size was:

$$\begin{aligned} AN = 2.025. \end{aligned}$$

In steady-state operation, two forklifts are actively used. The corresponding utilization level of two forklifts can therefore be expressed as:

$$\begin{aligned} U = \frac{WL}{2 \cdot AT} = \frac{29.766}{2 \cdot 14.7} = 1.012. \end{aligned}$$

This corresponds to an effective utilization of approximately $101.2\%$, indicating that two forklifts operate at near-full capacity under the specified steady-state demand. This explains the practical necessity of maintaining a third unit as backup to ensure operational robustness and service continuity in the presence of minor disturbances.

Considering the rounded fleet-size recommendation:

$$\begin{aligned} AN_r = \lceil 2.025 \rceil = 3, \end{aligned}$$

and the fact that three forklifts are available at the facility, the relative deviation between the analytically recommended discrete fleet size and the deployed fleet is:

$$\begin{aligned} \delta = \frac{|3 - 3|}{3} = 0. \end{aligned}$$

This indicates full agreement between the analytical fleet-sizing recommendation and the implemented industrial configuration. While the model does not claim formal optimality, the close quantitative correspondence demonstrates that the deterministic steady-state formulation provides a reliable approximation of effective fleet requirements under real industrial conditions.

#### Demand scaling scenario analysis

To further examine the quantitative behavior of the analytical formulation without relying on simulation-based validation, a structured demand-scaling scenario was evaluated. Since the required number of agents is directly proportional to the transport demand *w*, a proportional increase in all flow matrix elements results in a proportional increase in workload:

$$\begin{aligned} WL \propto w. \end{aligned}$$

Two demand growth scenarios were considered.

**Scenario 1 (+20% demand increase)**

If all material flows increase by 20%, then:

$$\begin{aligned} w_{\mathrm{+20\%}} = 1.2 \cdot 10.222 = 12.266 \ \text {pallets/hour}. \end{aligned}$$

The cycle time $T_C$ remains unchanged, therefore:

$$\begin{aligned} WL_{\mathrm{+20\%}} = 1.2 \cdot 29.77 = 35.72 \ \text {min/hour}. \end{aligned}$$

The required fleet size becomes:

$$\begin{aligned} AN_{\mathrm{+20\%}} = \frac{35.72}{14.7} = 2.43. \end{aligned}$$

Rounding yields:

$$\begin{aligned} AN_{r,\mathrm{+20\%}} = 3. \end{aligned}$$

**Scenario 2 (+50% demand increase)**

For a 50% demand increase:

$$\begin{aligned} w_{\mathrm{+50\%}} = 1.5 \cdot 10.222 = 15.333 \ \text {pallets/hour}. \end{aligned}$$

$$\begin{aligned} WL_{\mathrm{+50\%}} = 1.5 \cdot 29.77 = 44.66 \ \text {min/hour}. \end{aligned}$$

$$\begin{aligned} AN_{\mathrm{+50\%}} = \frac{44.66}{14.7} = 3.04. \end{aligned}$$

Rounding yields:

$$\begin{aligned} AN_{r,\mathrm{+50\%}} = 4. \end{aligned}$$

The results confirm the linear scaling behavior predicted by the analytical formulation. The model exhibits stable and monotonic fleet growth under increasing demand conditions, supporting its applicability for early-stage capacity planning and production scaling decisions.

#### Analytical error bound assessment

Since the required number of agents is given by

$$\begin{aligned} AN = \frac{w T_C}{60 A F_t E_w}, \end{aligned}$$

the model exhibits inverse proportional sensitivity to the aggregated operational factor

$$\begin{aligned} \Phi = A F_t E_w. \end{aligned}$$

For small perturbations, the relative variation of the fleet size can be expressed as

$$\begin{aligned} \frac{\Delta AN}{AN} = -\frac{\Delta \Phi }{\Phi }. \end{aligned}$$

Assuming a conservative uncertainty of $\pm 5\%$ in each operational parameter (*A*, $F_t$, $E_w$), the combined worst-case deviation of the aggregated factor is approximately

$$\begin{aligned} \Phi _{min} = 0.95^3 \Phi \approx 0.857 \Phi . \end{aligned}$$

The corresponding upper bound of fleet size becomes

$$\begin{aligned} AN_{max} = \frac{AN}{0.857} \approx 1.167 AN. \end{aligned}$$

The result is:

$$\begin{aligned} AN = 2.025 \Rightarrow AN_{max} \approx 2.36. \end{aligned}$$

This value remains below the rounding threshold of 3 agents. Therefore, moderate parameter estimation uncertainties do not affect the discrete fleet-sizing recommendation.

### Example 2 - SMARTHam

#### Industrial context and inspection task

The second case study was conducted within the SMARTHam KTE at Capanna Prosciutti a producer of Parma ham. The objective was to determine the required number of AGVs for warehouse inventory inspection. Each ham was equipped with an RFID tag. An AGV equipped with an RFID antenna performed periodic inspection by traversing predefined routes through the warehouse and reading tags on storage racks. Unlike Example 1, this case represents an inspection task rather than material transport.

**Fig. 3:**
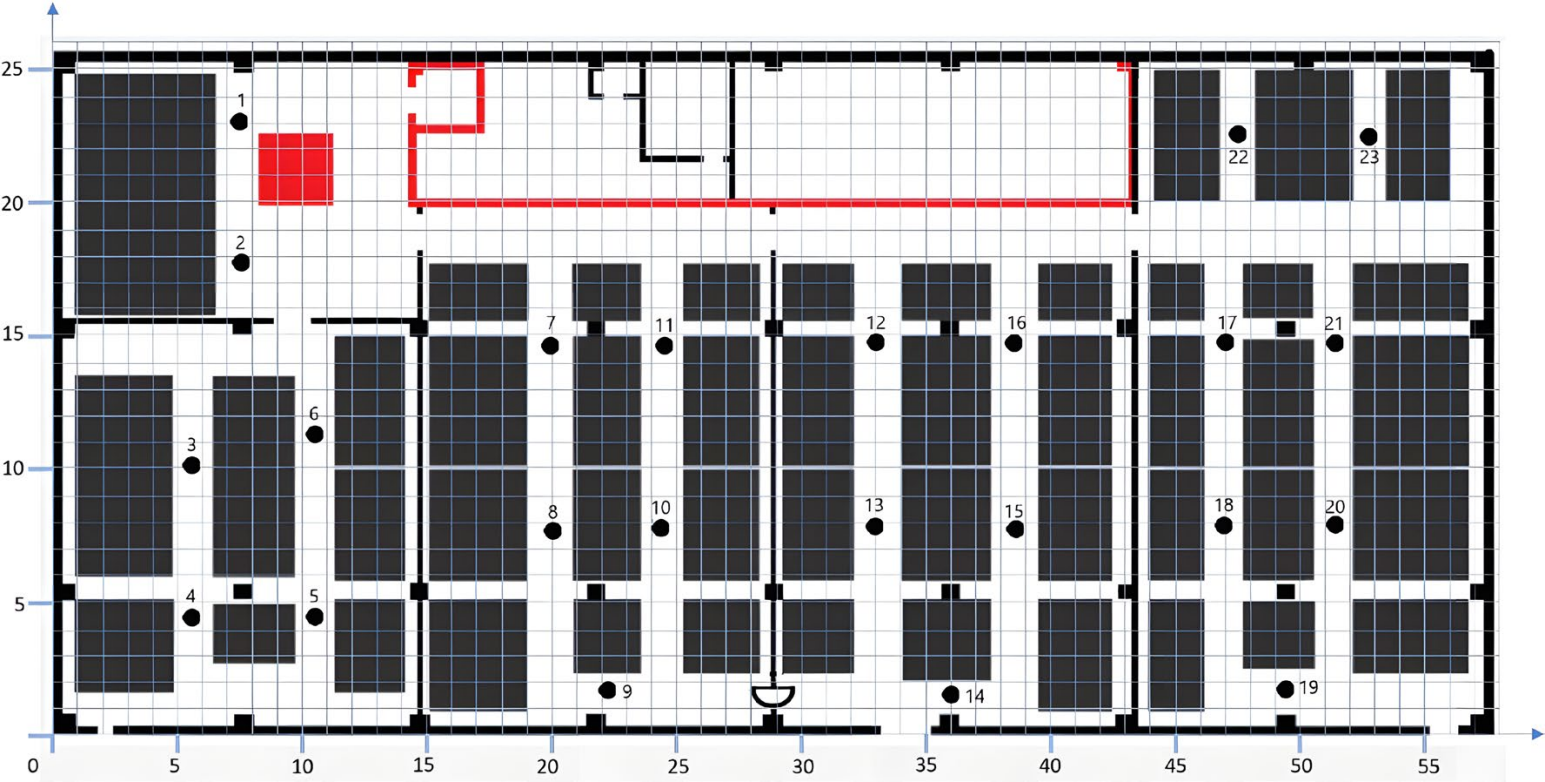
Sketch of the factory (machines and routes between stations).

The 2D factory layout is shown in Fig. 3. Numerical labels correspond to station identifiers and shaded regions indicate obstacles such as walls and storage racks used for ham storage during inspection. A single cyclic inspection route covering all stations was defined. The flow rate matrix (Tab. 3) represents inspection frequency per hour, while the distance matrix (Tab. 4) defines travel distances in meters.

The analysis was performed under steady-state assumptions with hourly averaged inspection requirements.

#### Input data and operational parameters

The AGV parameters were:

- Agent speed: $v_c = 65$ m/min
- Loading time: $T_L = 0.0001$ min (negligible)
- Unloading time (inspection time): $T_U = 1.0$ min
- Capacity: $c = 23$ stations per cycle
- Availability: $$A = 0.99$$
- Traffic factor: $$F_t = 0.99$$
- Operator efficiency: $$E_w = 1.0$$

All calculations are performed per hour. The capacity $c=23$ reflects the number of stations inspected within one complete route before returning to the starting point.

#### Analytical fleet sizing using proposed model

The intermediate derived quantities representing delivery cycles were:

$$\begin{aligned} w \approx 22.0 \ \text {cycles/hour}, \quad L_d = 52.6 \ \text {m}, \quad L_e = 52.0 \ \text {m}. \end{aligned}$$

Using the methodology described in Section "Agent Sizing Method", the average cycle time per inspection cycle was computed as:

$$\begin{aligned} T_C = 1.807 \ \text {min}. \end{aligned}$$

The total workload per hour is therefore:

$$\begin{aligned} WL = 39.76 \ \text {min/hour}. \end{aligned}$$

The effective available time per AGV per hour is:

$$\begin{aligned} AT = 60 \cdot A \cdot F_t \cdot E_w = 60 \cdot 0.99 \cdot 0.99 \cdot 1.0 = 58.806 \ \text {min/hour}. \end{aligned}$$

The required number of AGVs is:

$$\begin{aligned} AN = \frac{WL}{AT} = \frac{39.76}{58.806} = 0.676. \end{aligned}$$

Rounding up yields:

$$\begin{aligned} AN_r = 1. \end{aligned}$$

Thus, a single AGV is sufficient to perform the inspection task within the required hourly frequency.

#### Baseline comparison with classical analytical fleet sizing

Without correction factors ($A = F_t = E_w = 1$):

$$\begin{aligned} AT_{baseline} = 60 \ \text {min/hour}, \end{aligned}$$

$$\begin{aligned} AN_{baseline} = \frac{39.76}{60} = 0.663. \end{aligned}$$

Because availability and traffic factors are close to unity in this case, the baseline and proposed model yield nearly identical results. This confirms that the correction factors primarily influence fleet size when operational inefficiencies are significant.

#### Sensitivity analysis

To evaluate robustness, key parameters were varied individually while keeping the remaining parameters constant.

**Traffic factor variation**

- $$F_t = 0.9$$ $$\Rightarrow$$ $$AT = 53.46$$ min/hour $$\Rightarrow$$ $$AN = 0.744$$
- $$F_t = 0.8$$ $$\Rightarrow$$ $$AT = 47.52$$ min/hour $$\Rightarrow$$ $$AN = 0.837$$

Even with substantial congestion effects, the required number of agents remains below 1, and rounding still results in a single AGV.

**Availability variation**

- $$A = 0.9$$ $$\Rightarrow$$ $$AT = 53.46$$ min/hour $$\Rightarrow$$ $$AN = 0.744$$
- $$A = 0.8$$ $$\Rightarrow$$ $$AT = 47.52$$ min/hour $$\Rightarrow$$ $$AN = 0.837$$

The proportional relationship between *AN* and $1/(A F_t E_w)$ is preserved, and the model response remains stable.

**Speed variation** ($$\pm 10\%$$)

The cycle time is given by:

$$\begin{aligned} T_C = T_L + T_U + \frac{L_d + L_e}{v_c}. \end{aligned}$$

Only the transport component depends on $v_c$, while handling times remain constant.

- $v_c = 58.5$ m/min ($-10\%$) $\Rightarrow$ $T_C \approx 1.89$ min $\Rightarrow$ $WL \approx 41.6$ min/hour $\Rightarrow$ $AN \approx 0.707$
- $v_c = 71.5$ m/min ($+10\%$) $\Rightarrow$ $T_C \approx 1.74$ min $\Rightarrow$ $WL \approx 38.0$ min/hour $\Rightarrow$ $AN \approx 0.646$

The relationship is monotonic and consistent with the analytical formulation. Moderate speed variations do not cause threshold instability.

$$\begin{aligned} AN \propto \frac{1}{A F_t E_w}. \end{aligned}$$

The rounding boundary lies at $AN = 1$. Even under conservative parameter assumptions, the analytical result consistently supports a single AGV solution.

#### Operational confirmation

In practice, a single AGV was deployed for warehouse inspection. The analytical prediction ($AN = 0.676$, rounded to 1) matches the observed operational deployment. No additional AGVs were required to meet inspection frequency requirements. The result is therefore operationally consistent with real industrial conditions.

#### Quantitative consistency assessment

To further examine the quantitative consistency of the analytical estimation with observed deployment, a utilization-based assessment was performed. The analytically predicted continuous fleet size was:

$$\begin{aligned} AN = 0.676. \end{aligned}$$

In practice, one AGV is deployed for warehouse inspection. The corresponding utilization level can therefore be expressed as:

$$\begin{aligned} U = \frac{WL}{AT} = \frac{39.76}{58.806} = 0.676. \end{aligned}$$

This corresponds to an effective utilization of approximately $67.6\%$, indicating that the deployed AGV operates with a stable capacity margin under steady-state inspection requirements. Considering the rounded fleet-size recommendation:

$$\begin{aligned} AN_r = \lceil 0.676 \rceil = 1, \end{aligned}$$

the relative deviation between the analytically recommended discrete fleet size and the observed deployment is:

$$\begin{aligned} \delta = \frac{|1 - 1|}{1} = 0. \end{aligned}$$

This indicates full agreement between the analytical fleet-sizing recommendation and the implemented industrial solution. While the model does not claim formal optimality, the quantitative correspondence demonstrates that the deterministic steady-state formulation yields consistent and practically applicable fleet-size estimates under the specified operating conditions.

#### Demand scaling scenario analysis

To further evaluate the analytical behavior of the inspection model, a proportional demand-scaling scenario was examined. Because the required number of agents is directly proportional to the inspection demand *w*, a uniform scaling of all flow matrix elements results in a proportional scaling of workload:

$$\begin{aligned} WL \propto w, \qquad AN \propto w. \end{aligned}$$

The cycle time $T_C$ and available time *AT* remain unchanged under proportional scaling, since average travel distances per delivery and operational parameters are unaffected.

**Scenario 1 (+20% inspection demand)**

$$\begin{aligned} WL_{\mathrm{+20\%}} = 1.2 \cdot 39.76 = 47.71 \ \text {min/hour}. \end{aligned}$$

$$\begin{aligned} AN_{\mathrm{+20\%}} = 1.2 \cdot 0.676 = 0.811. \end{aligned}$$

Rounding yields:

$$\begin{aligned} AN_{r,\mathrm{+20\%}} = 1. \end{aligned}$$

**Scenario 2 (+50% inspection demand)**

$$\begin{aligned} WL_{\mathrm{+50\%}} = 1.5 \cdot 39.76 = 59.64 \ \text {min/hour}. \end{aligned}$$

$$\begin{aligned} AN_{\mathrm{+50\%}} = 1.5 \cdot 0.676 = 1.014. \end{aligned}$$

Rounding yields:

$$\begin{aligned} AN_{r,\mathrm{+50\%}} = 2. \end{aligned}$$

The results confirm the linear scaling behavior of the analytical formulation. Under moderate demand growth (+20%), a single AGV remains sufficient. A substantial demand increase (+50%) leads to a transition across the rounding threshold, requiring a second AGV. This predictable and monotonic behavior supports the suitability of the deterministic steady-state formulation for capacity planning and inspection-frequency scaling.

#### Analytical error bound assessment

Applying the analytical robustness bound introduced in Section “Examples” for Example 1 and assuming a conservative $\pm 5\%$ uncertainty in the operational parameters *A*, $F_t$, and $E_w$, the upper bound of the fleet size becomes:

$$\begin{aligned} AN = 0.676 \Rightarrow AN_{max} \approx 0.789. \end{aligned}$$

This value remains below the rounding threshold of one agent. Therefore, moderate parameter estimation uncertainties do not affect the final discrete fleet-sizing recommendation.

## Conclusion

This study presented a deterministic analytical fleet-sizing method for estimating the required number of logistics agents under steady-state operating conditions. The proposed approach extends a classical analytical formulation by incorporating matrix-based representations of material flows and distances together with operational correction factors reflecting availability, traffic influence and human efficiency. The method does not formulate a formal optimization problem with explicit objective functions and constraints, rather, it provides a structured and transparent capacity estimation framework suitable for early-stage logistics planning and system scaling.

The methodology was implemented as an independent Docker-based service and evaluated through two industrial case studies within the Better Factory project. In both cases, the analytically estimated fleet size was consistent with observed operational deployment. The validation does not rely solely on post-hoc agreement with observed deployment, but is further supported by consistency analysis, utilization-based evaluation, demand scaling scenarios and analytical error bounds, which together demonstrate robustness of the proposed formulation under varying operating conditions.

The proposed approach operates under clearly defined assumptions, including steady-state deterministic flows, predefined routes and aggregated representation of operational variability. It is therefore intended primarily as a capacity planning and decision-support tool rather than a substitute for detailed dynamic simulation. In systems characterized by highly stochastic demand patterns, complex routing conflicts or queueing phenomena, complementary simulation-based validation may be appropriate.

Future work will focus on extending the framework in several directions. Incorporation of stochastic flow models and variability analysis will enhance applicability in non-steady-state environments for real time demands. Integration with broader digital twin architectures may allow hybrid analytical–simulation workflows that combine computational efficiency with dynamic accuracy.

Overall, the presented analytical method contributes a transparent and accessible fleet-sizing tool that can support logistics planning decisions, particularly in SMEs where interpretability, ease of deployment and reproducibility are critical.

## Supplementary Information

Supplementary Information.

## Data Availability

All data supporting the findings of this study are available within the article itself.

## Abbreviations

OPIL: Open Platform for Innovation in Logistics
AGV: Automated Guided Vehicle
ANN: Artificial Neural Networks
RSM: Response Surface Methodology
MLR: Multiple Linear Regression
ERP: Enterprise Resource Planning
ROS: Robot Operating System
NGSI: Next Generation Service Interface
SME: Small and Medium-sized Enterprises
IoT: Internet of Things
OCB: Orion Context Broker
HMI: Human-Machine Interface
SP: Sensing & Perception
RAN: Robot Agent Node
KTE: Knowledge Transfer Experiment
RFID: Radio Frequency Identification

## Code availability

The source code of the Agent Optimization module is publicly available at: https://github.com/ramp-eu/Agent_Optimisation_Service. Additional information about the application is available at: https://betterfactory.eu/apps/agent-optimization.
