# Supplementary material for: Analytical fleet-sizing method for the Open Platform for Innovation in Logistics (OPIL)
Source: Sci Rep. 2026 Apr 9;16:16797. doi: 10.1038/s41598-026-46148-y (PMC13223205; doi:10.1038/s41598-026-46148-y)
Supplement: Supplementary file 1 — Supplementary Information. [file 41598_2026_46148_MOESM1_ESM.pdf]

ST1	ST2	ST3	ST4	ST5	ST6	ST7	ST8	ST9	ST10	ST11	ST12	ST13	ST14	ST15	ST16	ST17	ST18
0	0	0	0	0	0	0	0	0	0	0	0	0	0	0	0	0.15	0
0	0	0	0	0	0	0	0	0	0	0	0	0	0	0	0	0.312	0
0	0	0	0	0	0	0	0	0	0	0	0	0	0	0	0	0.25	0
0	0	0	0	0	0	0	0	0	0	0	0	0	0	0	0	0.312	0
0	0	0	0	0	0	0	0	0	0	0	0	0	0	0	0	0.625	0
0	0	0	0	0	0	0	0	0	0	0	0	0	0	0	0	0.188	0
0	0	0	0	0	0	0	0	0	0	0	0	0	0	0	0	0.15	0
0	0	0	0	0	0	0	0	0	0	0	0	0	0	0	0	0.15	0
0	0	0	0	0	0	0	0	0	0	0	0	0	0	0	0	0.15	0
0	0	0	0	0	0	0	0	0	0	0	0	0	0	0	0	0.125	0
0	0	0	0	0	0	0	0	0	0	0	0	0	0	0	0	0.188	0
0	0	0	0	0	0	0	0	0	0	0	0	0	0	0	0	0.125	0
0	0	0	0	0	0	0	0	0	0	0	0	0	0	0	0	0.162	0
0	0	0	0	0	0	0	0	0	0	0	0	0	0	0	0	0.162	0
0	0	0	0	0	0	0	0	0	0	0	0	0	0	0	0	0.312	0
0	0	0	0	0	0	0	0	0	0	0	0	0	0	0	0	0.312	0
0	0	0	0	0	0	0	0	0	0	0	0	0	0	0	0	0	3.675
0.125	0.25	0.25	0.25	0.375	0.125	0.125	0.125	0.125	0.125	0.125	0.125	0.125	0.125	0.25	0.25	0	0

Table 1. Flow Rate Matrix

ST1	ST2	ST3	ST4	ST5	ST6	ST7	ST8	ST9	ST10	ST11	ST12	ST13	ST14	ST15	ST16	ST17	ST18
0	0	0	0	0	0	0	0	0	0	0	0	0	0	0	0	71	0
0	0	0	0	0	0	0	0	0	0	0	0	0	0	0	0	69	0
0	0	0	0	0	0	0	0	0	0	0	0	0	0	0	0	67	0
0	0	0	0	0	0	0	0	0	0	0	0	0	0	0	0	75	0
0	0	0	0	0	0	0	0	0	0	0	0	0	0	0	0	87	0
0	0	0	0	0	0	0	0	0	0	0	0	0	0	0	0	68	0
0	0	0	0	0	0	0	0	0	0	0	0	0	0	0	0	79	0
0	0	0	0	0	0	0	0	0	0	0	0	0	0	0	0	60	0
0	0	0	0	0	0	0	0	0	0	0	0	0	0	0	0	40	0
0	0	0	0	0	0	0	0	0	0	0	0	0	0	0	0	24	0
0	0	0	0	0	0	0	0	0	0	0	0	0	0	0	0	20	0
0	0	0	0	0	0	0	0	0	0	0	0	0	0	0	0	46	0
0	0	0	0	0	0	0	0	0	0	0	0	0	0	0	0	18	0
0	0	0	0	0	0	0	0	0	0	0	0	0	0	0	0	27	0
0	0	0	0	0	0	0	0	0	0	0	0	0	0	0	0	25	0
0	0	0	0	0	0	0	0	0	0	0	0	0	0	0	0	30	0
0	0	0	0	0	0	0	0	0	0	0	0	0	0	0	0	0	80
83	83	80	88	100	79	92	74	56	41	61	61	61	70	71	79	0	0

Table 2. Distance Matrix

ST1	ST2	ST3	ST4	ST5	ST6	ST7	ST8	ST9	ST10	ST11	ST12	ST13	ST14	ST15	ST16	ST17	ST18	ST19	ST20	ST21	ST22	ST23
0	1	0	0	0	0	0	0	0	0	0	0	0	0	0	0	0	0	0	0	0	0	0
0	0	1	0	0	0	0	0	0	0	0	0	0	0	0	0	0	0	0	0	0	0	0
0	0	0	1	0	0	0	0	0	0	0	0	0	0	0	0	0	0	0	0	0	0	0
0	0	0	0	1	0	0	0	0	0	0	0	0	0	0	0	0	0	0	0	0	0	0
0	0	0	0	0	1	0	0	0	0	0	0	0	0	0	0	0	0	0	0	0	0	0
0	0	0	0	0	0	1	0	0	0	0	0	0	0	0	0	0	0	0	0	0	0	0
0	0	0	0	0	0	0	1	0	0	0	0	0	0	0	0	0	0	0	0	0	0	0
0	0	0	0	0	0	0	0	1	0	0	0	0	0	0	0	0	0	0	0	0	0	0
0	0	0	0	0	0	0	0	0	1	0	0	0	0	0	0	0	0	0	0	0	0	0
0	0	0	0	0	0	0	0	0	0	1	0	0	0	0	0	0	0	0	0	0	0	0
0	0	0	0	0	0	0	0	0	0	0	1	0	0	0	0	0	0	0	0	0	0	0
0	0	0	0	0	0	0	0	0	0	0	0	1	0	0	0	0	0	0	0	0	0	0
0	0	0	0	0	0	0	0	0	0	0	0	0	1	0	0	0	0	0	0	0	0	0
0	0	0	0	0	0	0	0	0	0	0	0	0	0	1	0	0	0	0	0	0	0	0
0	0	0	0	0	0	0	0	0	0	0	0	0	0	0	1	0	0	0	0	0	0	0
0	0	0	0	0	0	0	0	0	0	0	0	0	0	0	0	1	0	0	0	0	0	0
0	0	0	0	0	0	0	0	0	0	0	0	0	0	0	0	0	1	0	0	0	0	0
0	0	0	0	0	0	0	0	0	0	0	0	0	0	0	0	0	0	1	0	0	0	0
0	0	0	0	0	0	0	0	0	0	0	0	0	0	0	0	0	0	0	1	0	0	0
0	0	0	0	0	0	0	0	0	0	0	0	0	0	0	0	0	0	0	0	1	0	0
0	0	0	0	0	0	0	0	0	0	0	0	0	0	0	0	0	0	0	0	0	1	0
0	0	0	0	0	0	0	0	0	0	0	0	0	0	0	0	0	0	0	0	0	0	1
-1	0	0	0	0	0	0	0	0	0	0	0	0	0	0	0	0	0	0	0	0	0	0

Table 3. Flow rate Matrix

ST1	ST2	ST3	ST4	ST5	ST6	ST7	ST8	ST9	ST10	ST11	ST12	ST13	ST14	ST15	ST16	ST17	ST18	ST19	ST20	ST21	ST22	ST23
0	5	0	0	0	0	0	0	0	0	0	0	0	0	0	0	0	0	0	0	0	0	0
0	0	13	0	0	0	0	0	0	0	0	0	0	0	0	0	0	0	0	0	0	0	0
0	0	0	6	0	0	0	0	0	0	0	0	0	0	0	0	0	0	0	0	0	0	0
0	0	0	0	11	0	0	0	0	0	0	0	0	0	0	0	0	0	0	0	0	0	0
0	0	0	0	0	7	0	0	0	0	0	0	0	0	0	0	0	0	0	0	0	0	0
0	0	0	0	0	0	23	0	0	0	0	0	0	0	0	0	0	0	0	0	0	0	0
0	0	0	0	0	0	0	7	0	0	0	0	0	0	0	0	0	0	0	0	0	0	0
0	0	0	0	0	0	0	0	8	0	0	0	0	0	0	0	0	0	0	0	0	0	0
0	0	0	0	0	0	0	0	0	8	0	0	0	0	0	0	0	0	0	0	0	0	0
0	0	0	0	0	0	0	0	0	0	7	0	0	0	0	0	0	0	0	0	0	0	0
0	0	0	0	0	0	0	0	0	0	0	16	0	0	0	0	0	0	0	0	0	0	0
0	0	0	0	0	0	0	0	0	0	0	0	7	0	0	0	0	0	0	0	0	0	0
0	0	0	0	0	0	0	0	0	0	0	0	0	10	0	0	0	0	0	0	0	0	0
0	0	0	0	0	0	0	0	0	0	0	0	0	0	9	0	0	0	0	0	0	0	0
0	0	0	0	0	0	0	0	0	0	0	0	0	0	0	7	0	0	0	0	0	0	0
0	0	0	0	0	0	0	0	0	0	0	0	0	0	0	0	16	0	0	0	0	0	0
0	0	0	0	0	0	0	0	0	0	0	0	0	0	0	0	0	7	0	0	0	0	0
0	0	0	0	0	0	0	0	0	0	0	0	0	0	0	0	0	0	9	0	0	0	0
0	0	0	0	0	0	0	0	0	0	0	0	0	0	0	0	0	0	0	8	0	0	0
0	0	0	0	0	0	0	0	0	0	0	0	0	0	0	0	0	0	0	0	7	0	0
0	0	0	0	0	0	0	0	0	0	0	0	0	0	0	0	0	0	0	0	0	12	0
0	0	0	0	0	0	0	0	0	0	0	0	0	0	0	0	0	0	0	0	0	0	12
52	0	0	0	0	0	0	0	0	0	0	0	0	0	0	0	0	0	0	0	0	0	0

Table 4. Distance Matrix
